# Effect of Core Balance Training on Muscle Tone and Balance Ability in Adult Men and Women

**DOI:** 10.3390/ijerph191912190

**Published:** 2022-09-26

**Authors:** Sun-Ha Jo, Hyuk-Jae Choi, Hyeon-Seok Cho, Jin-Hwan Yoon, Won-Young Lee

**Affiliations:** 1 Department of Advanced industry Convergence, Chosun University, Gwangju 61452, Korea; 2 Department of Medical Convergence Research & Development, Rehabilitation Engineering Research Institute, Korea Workers’ Compensation & Welfare Service, Incheon 21419, Korea; 3 Institute of Sports Medicine, Hannam University, Daejeon 34430, Korea

**Keywords:** core training, muscle tone, muscle stiffness, balance ability, Myoton PRO

## Abstract

(1) Background: The amount of physical activity most adults perform is less than the recommended amount, and the resulting decrease in physical strength makes them vulnerable to various diseases. A decrease in muscle size and strength due to damage caused by disease or aging negatively affects functional strength. Muscle evaluation in adults can yield results that are predictive indicators of aging and unexpected disability. In addition, balance ability is essential to prevent falls and injuries in daily life and maintain functional activities. It is important to develop and strengthen balance in the lower extremities and core muscles to maintain and enhance overall body balance. This study aimed to analyze the effects of core balance training on muscle tone and balance ability in adults. (2) Methods: The participants of this study were 32 adult male and female university students (male: mean age = 21.3 ± 1.9 years, weight = 74.2 ± 12.6 kg, BMI = 23.4 + 2.5, *n* = 14; female: mean age = 21.0 ± 1.4 years, weight = 64.6 + 1.2 kg, BMI = 22.4 ± 2.4, *n* =18). Thirty-two adults (training group: 16, control group: 16; male: 16, female: 16) participated in the Myoton PRO (gastrocnemius lateral/medial, tibialis anterior), Pedalo balance system, and Y-balance test. (3) Results: The following results were obtained for muscle elasticity, stiffness, and dynamic/static balance ability after 10 weeks of core balance training. 1. There was no significant difference in muscle elasticity (gastrocnemius lateral/medial, tibialis anterior) (*p* < 0.05). 2. Muscle stiffness (gastrocnemius lateral/medial, tibialis anterior) significantly increased (*p* < 0.05). 3. Dynamic/static balance ability significantly increased (*p* < 0.05). (4) Conclusions: In future, data for the age and sex of various participants, should be accumulated by recruiting participants to study muscle characteristics, such as muscle elasticity and stiffness. Estimating the appropriate injury range and optimal exercise capacity is possible through follow-up studies. The findings can then be used as a basis for predicting injuries or determining and confirming the best time to resume exercise.

## 1. Introduction

Modern society is progressing to a high level of industrialization. However, as society becomes more industrialized, it fails to attain the recommended levels of physical activity [1]. Most adults' physical activity fails to comply with WHO recommendations, and the subsequent decline in physical strength makes modern people vulnerable to various diseases [2]. Health can be preserved by emphasizing an active daily lifestyle as the average human lifespan has increased [3]. Factors that have recently attracted attention for maintaining a healthy daily life include muscle condition and body balance ability [4].

Studies have shown that muscle size and strength are decreased owing to damage from physical activity, disease, or aging [5], and a decrease in muscle strength with age has a negative effect on functional strength, such as balance ability [6]. In addition, since the musculoskeletal system undergoes a period of quantitative and qualitative muscle development in individuals’ 30s [7], muscle evaluation in adults can yield results that are predictive indicators of aging and unexpected disability [8].

Muscle tone is classified according to the measuring equipment and purpose. Currently, the methods mainly used to evaluate muscle condition include isokinetic exercise, muscle activity (EMG), and muscle tone (TMG) [9]. Factors that can be checked using the equipment currently used in rehabilitation medicine include muscle tone, stiffness, elasticity, recovery, and elongation [10,11,12,13]. However, research on muscle elasticity properties conducted to date has only targeted patients with muscle disease or the elderly [14,15,16,17]; it is necessary to expand the scope of studies to various individuals, including adults. In addition, a study on the lower extremities along with large muscles, such as the quadriceps, is required for the overall and balanced development of the lower body.

Meanwhile, balance ability is a major variable involved in daily movements and gait. It has a high correlation with functional performance [18]. Therefore, maintaining body balance in daily life is essential for preventing falls and injuries and maintaining functional activities. In summary, balance ability is essential to lead an active daily life. The balanced development and strengthening of lower extremities and core muscles is important to maintain and promote balance ability [19]. Accordingly, integrated relationship analysis and interaction analysis between sub-factors are required to study variables related to balance ability.

Considering duration and intensity, it is hypothesized that core exercise can change body composition, muscle properties, and balance ability in adults in their 20s. To date, research on core balance training has been widely conducted, including on the sport of athletes, research based on age—such as among young and elderly individuals, and research on training program development [20,21,22]. However, most studies compared differences in the effect according to time through pre-and post-testing, and continuous effect tracking was not performed. Muscle evaluation in adults can confirm the possibility of injury and be a predictive index of disability according to age.

Therefore, the purpose of this study was to present a new horizon for balance ability by verifying the effect of core balance training on healthy men and women in their 20s and the correlation between muscle characteristics and balance ability [10,11].

## 2. Materials and Methods

### 2.1. Subject and Core Balance Training

This study included 32 adult male and female university students (male: mean age = 21.3 ± 1.9 years, weight = 74.2 ± 12.6 kg, BMI = 23.4 + 2.5, *n* = 14; female: mean age = 21.0 ± 1.4 years, weight = 64.6 + 1.2 kg, BMI = 22.4 ± 2.4, *n* = 18). The study subjects were active [23], healthy adults with no muscle-related injuries within the last 3 years and had not participated in any other exercise program within 6 months (Table 1).

**Figure 1:**
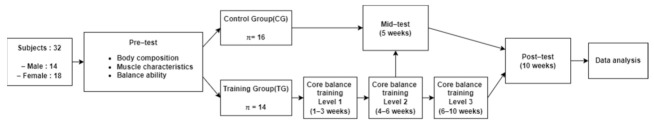
Research design.

The study design is illustrated in Figure 1. Core balance training was conducted for 10 weeks, and the subjects were restricted from participating in other exercises during the training period. The training was divided into three stages such that the subject could adapt to the changing training difficulty. Core balance training was conducted for 1h, 2 days each week, excluding weekends, and the intensity of the training was gradually increased at intervals of 3 weeks (Table 2). During training, the main movements included in the core balance training program consisted of a squat movement to strengthen the lower extremities and glutes [24], a leg raise for strengthening the abdominal core muscles [25], and a plank movement for strengthening the overall core muscles [26].

This study was approved by the Institutional Review Board (No. RERI-IRB-210416) of the Rehabilitation Engineering Research Institute. After receiving a complete description of the study, consent was obtained from the study participants.

### 2.2. Body Composition and Muscle Elasticity and Stiffness 

Inbody 720 (Biospace Co, Seoul, Korea) was used to measure the participants' body weight (WGT), body fat (BF), and BMI. The participant removed metal accessories, took an upright position with arms and legs slightly apart, stood barefoot on the marked position of the measuring system, and held the electrode handle with the hand, and the body composition was analyzed.

The muscles to be measured were the bilateral tibialis anterior (TA), gastrocnemius medial (GM), and gastrocnemius lateral (GL) without discrimination on the dominant side. For the measurement of posture, the abdomen and chest were placed in a comfortable position, the supine and prone positions were alternated, and the elasticity and stiffness of the lower leg muscles were measured. The measurement location of the muscle elastic device probe was decided by selecting the two-thirds close to the lateral condyle of the tibia as the measurement location from the muscle between the tibia lateral condyle and the medial cuneiform bone in the case of TA. Measurement of the inner and outer gastrocnemius muscles was performed by plantar flexion, selecting the halfway point from the femoral head to the distal end of the gastrocnemius as the measurement location. During the measurement period, the measurement position was periodically marked using a marker to maintain the same measurement position. In this study, the decrement (Dec) value measured using Myoton PRO (MYOTON AS, Tallinn, Estonia) refers to the biomechanical properties of the muscle for its ability to recover its initial muscle shape after the reduction or removal of external force. Therefore, since muscle elasticity is inversely proportional to the Dec value, the lower the indicated value, the more elastic the muscle is. Stiffness (Stf) is the biomechanical property of a muscle that characterizes its resistance to contraction or external forces that deform its initial shape. All the experiments were conducted by an experienced anthropometrist.

### 2.3. Dynamic and Static Balance

The subjects' balance ability was measured using the Pedalo® Sensamove-Balance Test Pro (Sensamove, Groessen, Netherlands) and Y-balance method. Pedalo® Sensamove-Balance Test Pro measurement was performed by removing shoes, standing on a moving disk, maintaining balance, and performing balance movements according to the measurer's instructions, using the balance ability of the lower extremities. The Y-balance test was performed by pushing the prepared wooden box with the tip of the foot in the anterior, posterior, and posterolateral directions, using an instrument composed of the Y-balance test form on the floor. The sum of all measured values was divided by three times the participant's limb length, multiplied by 100, and divided into the left and right composite scores for the analysis

### 2.4. Statistics

To verify the training effect, pre-, mid-term, and post-tests were performed before training application, in the 5th week of training, and after training application, respectively. All data measured in this study were analyzed using the IBM SPSS Statistics for Windows, version 25(IBM Corp., Armonk, NY, USA). The difference between each measurement variable’s mean and standard deviation was verified by performing two-way repeated ANOVA, and the Scheffe method was used to analyze the differences according to the measurement group and measurement period. For all data, the statistical technique of the parametric method was used after the verification of normality and equal variance.

## 3. Results

### 3.1. Effects of Core Balance Training on Body Composition Index

**Figure 2:**
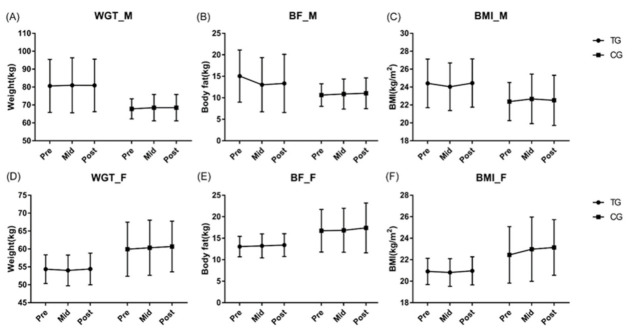
Effects of core balance training on body composition index. There was no effect of core training on body weight, WGT (**A**,**D**); body fat mass, BF (**B**,**E**); and BMI (**C**,**F**) in men and women. Abbreviations: WGT, body weight (kg); BF, body fat (kg); BMI, body mass index (kg/m^2^); M, male group; F, female group; Pre, pre-test; Mid, mid-test; Post, post-test; TG, training group; CG, control group.

Consequent to the analysis of changes in body composition index according to core balance training, no statistically significant differences were found between male (M) and female (F) body composition indexes between groups and measurement periods (Figure 2).

### 3.2. Difference between Muscle Elasticity and Muscle Stiffness according to Core Balance Training

**Figure 3:**
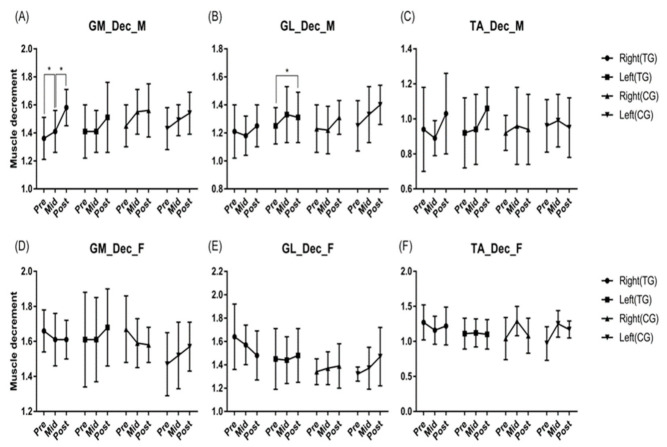
Effects of core balance training on muscle elasticity. There was difference according to time in men's right GM (*p* < 0.001) and left GL (*p* = 0.042). For women, there was a difference according to group time interaction in left GM (*p* = 0.045) and right TA (*p* = 0.027), and there was a difference according to group in right GL (*p* = 0.016). * *p* < 0.05. Abbreviations: GM, gastronemius medial; GL, gastronemius lateral; TA, tibialis anterior; Dec, muscle decrement; M, male group; F, female group; Pre, pre-test; Mid, mid-test; Post, post-test; TG, training group; CG, control group.

Based on the analysis of changes in muscle elasticity (Dec) according to core balance training, there was a difference according to time in the right GM and left GL of men (*p* < 0.05). For women, there was a difference according to group × time interaction in the left GM and right TA (*p* < 0.05), and there was a difference based on the group type in the right GL (*p* < 0.05) (Figure 3).

**Figure 4:**
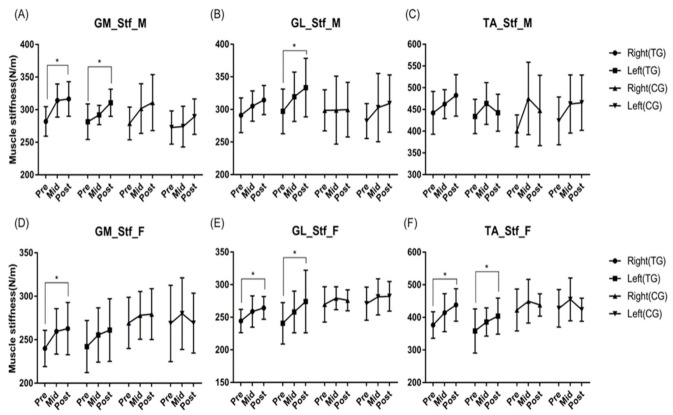
Effects of core balance training on muscle stiffness. There was a difference in muscle stiffness according to time in men's Right GM (*p* < 0.001), Left GM (*p* = 0.001) and Left GL (*p* = 0.003). For women, there was a difference according to time in Right GM (*p* = 0.001), Right GL (*p* = 0.001) and Left GL (*p* = 0.000), and Right TA (*p* = 0.043), and there was a difference according to group in Left TA (*p* = 0.024). * *p* < 0.05. Abbreviations: GM, gastronemius medial; GL, gastronemius lateral; TA, tibialis anterior; Stf, muscle stiffness (N/m); M, male group; F, female group; Pre, pre-test; Mid, mid-test; Post, post-test; TG, training group; CG, control group.

There was a difference in Stf according to time in men's right and left GM and left GL (p < 0.05). For women, there was a difference according to time in the right GM, right and left GL, and right TA (p < 0.05), and there was a difference based on the group type in the left TA (p < 0.05) (Figure 4).

### 3.3. Effect of Core Balance Training on Dynamic and Static Balance Ability

**Figure 5:**
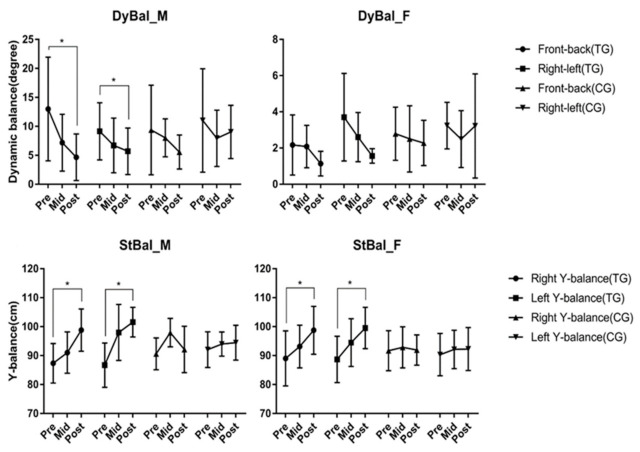
Effects of core balance training on Balance ability. There was a difference according to time in men's Front-Back (*p* = 0.004) and Right-Left (*p* = 0.039) in dynamic balance ability (DyBal). The static balance ability (StBal) showed a difference accord-ing to time in the male right (*p* = 0.003), left (p < 0.001) and group time interaction in right (*p* = 0.003). For women, there was a difference according to time in Right (*p* = 0.008)-Left (*p* = 0.000) and group time interaction in right (*p* = 0.003) and left (*p* = 0.003). * *p* < 0.05. Abbreviations: DyBal, dynamic balance ability; StBal, static balance ability; M, male group; F, female group; Pre, pre-test; Mid, mid-test; Post, post-test; TG, training group; CG, control group.

As a result of analyzing the change in dynamic balance ability (DyBal) according to core balance training, there was a difference according to time in men's front-back and right-left (*p* < 0.05). The static balance ability (StBal) showed a difference according to time in the male right–left (*p* < 0.05). For women, there was a difference according to time in right-left (*p* < 0.05) (Figure 5).

## 4. Discussion

This study aimed to analyze the effects of core balance training on muscle tone and balance ability in adults. The following results were obtained by measuring muscle elasticity, stiffness, and Dy/StBal after 10 weeks of core balance training. 1) There was no significant difference in muscle elasticity in the GL/GM and TA muscles (*p* < 0.05). 2) Muscle stiffness (GL/GM, TA) significantly increased (*p* < 0.05). 3) The Dy/StBal significantly increased (*p* < 0.05).

To verify the effect of core balance training on healthy adults in their 20s, this study examined changes in body composition, lower extremity muscle elasticity, muscle stiffness, and balance ability variables based on core balance training. The effects of core balance training known to date have a positive effect on various variables, such as improvement in muscle strength and balance ability [22]. Therefore, based on previous studies, the effect of core balance training conducted in this study is discussed further.

The change in elasticity and stiffness observed in the lower leg muscles in men is due to the negative correlation between elasticity and stiffness. As core balance training progresses, the balance control muscles of the lower extremities and muscle stiffness are strengthened, which contributes to a decrease in muscle elasticity [27]. The results showed that men and women had increased muscle stiffness during the study period. In particular, it was confirmed that the increase in gastrocnemius stiffness due to training was greater in men than in women.

To perform an exercise, the length of the muscle must change, and the elastic energy stored in the process of muscle contraction is used to express dynamic movement [28]. Therefore, muscle elasticity is importance for storing energy to produce movement. However, it is confirmed that the effect of core balance training on the elasticity of the lower leg muscles is negligible. A study with more samples is needed to explain the difference in the effect of training between men and women.

It is estimated that the improvement in muscle strength with core balance training is due to the improvement in muscle strength through training. Most studies analyzing the effect of core training on muscle function have focused on the quadriceps and hamstring; therefore, there are relatively few studies on the lower extremities [29]. When muscle stiffness is too low, there is a risk of muscle weakness; when it is too high, the risk of exercise injury due to reduced flexibility increases [30]. Therefore, an appropriate level of muscle stiffness is important to maintain health and prevent the risk of injury, and core balance training can be used as an effective method [12]. Meanwhile, studies on the elasticity and stiffness of muscles in terms of exercise-related injuries or patient exercise treatment are being conducted. However, there have been no studies on the lower extremities; therefore, presenting an appropriate level of muscle elasticity and range of muscle stiffness is difficult. In future, it is necessary to accumulate data through continuous research on the muscles of the lower extremities and their characteristics.

In a previous study, the training period was set to 8 weeks, and core training was conducted for a relatively short period. In contrast, when the subject was an elderly woman, a significant improvement in dynamic balance was reported after training [21]. This suggests that, even if the training target is an elderly person with a low level of basic physical strength, dynamic balance ability can be improved if training at an appropriate intensity is continuously performed.

Therefore, the training method of this study, which applied training for a certain period accompanied by a gradual increase in intensity, could improve the dynamic balance ability of adult men and women. Dynamic balance ability is the ability of the body to respond appropriately to gravity or external environmental factors to maintain equilibrium. It is manifested by comprehensively exerting motor nerves, muscle strength, and a sense of balance to maintain a stable posture [31,32]. In summary, the improvement in dynamic balance ability is due to the improvement in muscle strength and balance ability, which are the effects of core balance training.

An improvement in static balance ability because of core balance training has been confirmed in several studies [21,33,34,35]. Furthermore, core strengthening presents certain benefits; these data alone were insufficient to confirm dynamic balance’s effects on university judo athletes [36]. This may be because the detailed training program maintained the same intensity throughout the training period, unlike the present study. Therefore, the difference in the results may be attributed to the gradual increase in training intensity.

Although it is necessary to discuss further whether the increase in strength over time had an effect, the post-hoc analysis of this study confirmed the possibility of a continuous improvement in balance ability according to an increase in strength because static balance increases sequentially. Therefore, it is necessary to gradually increase load reinforcement or apply additional training to improve static balance ability in the composition of future core balance training.

## 5. Conclusions

From our discussion of previous studies, it is judged that the research on muscle elasticity and muscle stiffness is insufficient. These muscle-characteristics-related variables are currently used in rehabilitation-related medical institutions or research institutes, but the degree of academic research is insufficient. As a similar example, in the case of TMG, since a multifaceted approach to various participants has been continuously developed, it has been confirmed that some medical institutions are currently using it to diagnose and treat muscle injuries in athletes and patients [37,38]. The limitations of this study are as follows: (1). The research participants were limited to male and female adults enrolled in the university. (2). The participants' physical activities were not controlled. (3). The measurement site was limited to a specific lower extremity area. Further studies are needed to estimate the appropriate injury range and optimal exercise capacity. These findings can then be used as a basis for predicting injuries or determining and confirming the best time to resume exercise.

Therefore, if data on various subjects, such as age and sex, are accumulated through continuous research on muscle characteristics, such as muscle elasticity and muscle stiffness, injury prediction or exercise can be performed by estimating the appropriate range for injury and best exercise performance. This can be of practical help in the field of rehabilitation medicine, such as for confirming the time of resumption

## Figures and Tables

**Table 1:** Characteristics of subject.

		Training Group	Control Group	*p*-Value
Gender (*n*)	Male	7	7	
	Female	9	9	
Age (years)	Male	22.0 ± 2.07	20.7 ± 1.28	0.318
	Female	21.2 ± 0.79	20.44 ± 0.96	0.094
Weight (kg)	Male	80.63 ± 13.71	67.81 ± 5.18	0.128
	Female	54.3 ± 3.79	59.94 ± 7.13	0.258
Height (cm)	Male	182.4 ± 7.76	174.0 ± 5.29	0.097
	Female	161.2 ± 3.91	163.22 ± 3.19	0.113
Body mass index (kg/m^2^)	Male	24.4 ± 2.51	22.39 ± 1.97	0.165
	Female	23.5 ± 6.03	22.46 ± 2.49	0.436

Data presented as mean ± SD. Notes. All data values were verified as homogeneous groups (*p* > 0.05).

**Table 2:** Core balance training program.

Level	Target	Detail	Repetition
Level 1 (1–3 weeks)	Abdominals	Leg Raise	20 rep/2 set
		Flutter Kick	20 rep/2 set
		Both Heel Touch	20 rep/2 set
		V-Crunch	20 rep/2 set
		Plank	20 sec/2 set
	Waist	T-Figure	20 sec/2 set
		T-Fig, Touch toe	10 rep/2 set
	Hip	One Leg Lunge	20 rep/2 set
		Skater	20 rep/2 set
		Skater (Side·Back)	10 rep/2 set
		Skater Jump	20 rep/2 set
Level 2 (4–6 weeks)	Abdominals	Russian Twist	20 rep/2 set
		Heel Touch	20 rep/2 set
		Both Heel Touch	20 rep/2 set
		Cross Knee Touch	20 rep/2 set
		Plank	20 sec/2 set
	Waist	T-Figure	20 sec/2 set
		T-Fig, Touch toe	10 rep/2 set
		Glute Bridge	20 sec/2 set
		Bird Dog	10 rep/2 set
	Hip	Squat	20 rep/2 set
		Jump Skater	20 rep/2 set
		Skater	20 rep/2 set
		Skater Jump	20 rep/2 set
		One leg Lunge	10 rep/2 set
Level 3 (7–10 weeks)	Abdominals	Russian Twist	20 rep/2 set
		Heel Touch	20 rep/2 set
		Each Leg Raise	20 rep/2 set
		Leg Raise Touch Ankle	20 rep/2 set
		Hand on the Chest Sit-Up	20 rep/2 set
		Crunch	20 rep/2 set
		Cross Knee Touch	20 rep/2 set
	Waist	T-Figure	20 sec/2 set
		T-Fig, Touch Toe	10 rep/2 set
		Plank	20 sec/2 set
		Plank Leg Raise	10 rep/2 set
		Glute Bridge	10 sec/2 set
		Bird Dog	10 rep/2 set
	Hip	One leg Lunge	20 rep/2 set
		Side Lunge	20 rep/2 set
		Skater Jump	20 rep/2 set
		Squat	20 rep/2 set
		Jump Squat	20 rep/2 set

## Data Availability

The datasets used and analyzed during this study are available from the corresponding author on reasonable request.
